# Objective speech intelligibility prediction using a deep learning model with continuous speech-evoked cortical auditory responses

**DOI:** 10.3389/fnins.2022.906616

**Published:** 2022-08-18

**Authors:** Youngmin Na, Hyosung Joo, Le Thi Trang, Luong Do Anh Quan, Jihwan Woo

**Affiliations:** ^1^Department of Biomedical Engineering, University of Ulsan, Ulsan, South Korea; ^2^Department of Electrical, Electronic and Computer Engineering, University of Ulsan, Ulsan, South Korea

**Keywords:** speech intelligibility, deep-learning, continuous speech, occlusion sensitivity, EEG

## Abstract

Auditory prostheses provide an opportunity for rehabilitation of hearing-impaired patients. Speech intelligibility can be used to estimate the extent to which the auditory prosthesis improves the user’s speech comprehension. Although behavior-based speech intelligibility is the gold standard, precise evaluation is limited due to its subjectiveness. Here, we used a convolutional neural network to predict speech intelligibility from electroencephalography (EEG). Sixty-four–channel EEGs were recorded from 87 adult participants with normal hearing. Sentences spectrally degraded by a 2-, 3-, 4-, 5-, and 8-channel vocoder were used to set relatively low speech intelligibility conditions. A Korean sentence recognition test was used. The speech intelligibility scores were divided into 41 discrete levels ranging from 0 to 100%, with a step of 2.5%. Three scores, namely 30.0, 37.5, and 40.0%, were not collected. The speech features, i.e., the speech temporal envelope (ENV) and phoneme (PH) onset, were used to extract continuous-speech EEGs for speech intelligibility prediction. The deep learning model was trained by a dataset of event-related potentials (ERP), correlation coefficients between the ERPs and ENVs, between the ERPs and PH onset, or between ERPs and the product of the multiplication of PH and ENV (PHENV). The speech intelligibility prediction accuracies were 97.33% (ERP), 99.42% (ENV), 99.55% (PH), and 99.91% (PHENV). The models were interpreted using the occlusion sensitivity approach. While the ENV models’ informative electrodes were located in the occipital area, the informative electrodes of the phoneme models, i.e., PH and PHENV, were based on the occlusion sensitivity map located in the language processing area. Of the models tested, the PHENV model obtained the best speech intelligibility prediction accuracy. This model may promote clinical prediction of speech intelligibility with a comfort speech intelligibility test.

## Introduction

Auditory prostheses, such as hearing aids and cochlear implants, provide an excellent opportunity for hearing-impaired patients to rehabilitate their auditory modality. The outcome of auditory prosthesis use depends on the signal processing strategies: modulation of the current pulse train from sound in cochlear implants (CI) (Macherey et al., 2006; Wouters et al., 2015; Nogueira et al., 2019) or reduction of stationary and background noise and customized personal setting of hearing aids (Launer et al., 2016). In addition, the individual’s status, such as the insertion depth of the CI electrode, and the experience of cochlear implantation, could also affect the performance of CI (Vandali et al., 2000; Wanna et al., 2014). To evaluate the benefit of auditory prostheses, a behavioral speech intelligibility test is typically conducted using rating scales based on how well the listener comprehends sentences (Kim et al., 2009). In this behavioral test, a listener is asked to repeat or write what they hear in a recognition test. Speech intelligibility is estimated by scoring the number of correctly identified words (Enderby, 1980; Kent et al., 1989; Healy et al., 2015; Lee, 2016). Although the behavioral assessment can be conducted efficiently and quickly, a self-reported approach may be less reliable and less sensitive in evaluating the true hearing capability (Koelewijn et al., 2018). Vocoder simulation has also been used in speech tests to simulate the performance of hearing impairment in normal-hearing listeners (Mehta and Oxenham, 2017).

Event-related potentials (ERPs), in response to word or tone stimuli, have been used to evaluate auditory function objectively. Recently, several studies have shown that electroencephalography (EEG) signals in response to continuous speech stimuli are entrained to speech features: temporal envelope, spectrogram, and phonetics of speech (Scott et al., 2000; Liebenthal et al., 2005; Nourski et al., 2009; Ding and Simon, 2014; O’Sullivan et al., 2015; Crosse et al., 2016; Di Liberto et al., 2018). The speech temporal envelope (ENV), developed using the temporal response function model (TRF), is an effective feature to understand neural responses to continuous speech (Ciccarelli et al., 2019; Nogueira and Dolhopiatenko, 2020, 2022). However, the TRF model is limited in analyzing short (<5 s) responses due to the impact of onset response to a sentence (Crosse et al., 2016, 2021). Therefore, cross-correlation, which measures the similarity between the neural response and the speech sentence, can be more reliable in tracking neural signals in response to short sentences.

It was reported that speech intelligibility affected ENV entrainment (Ding and Simon, 2013; Vanthornhout et al., 2018; Lesenfants et al., 2019; Nogueira and Dolhopiatenko, 2022). Sentence comprehension requires complex hierarchical stages that integrate the phonological and prosodic processes of an acoustic input (Snedeker and Huang, 2009). Vanthornhout et al. (2018) developed a prediction model for the speech reception threshold using the TRF model, which could explain the variance of speech reception. Moreover, Di Liberto et al. (2015) showed that a speech prediction model with a phonetic feature was outperformed by the envelope model. Thus, a combination of ENV and phoneme (PH) onset information can be effective for feature computation. However, predicting a speech intelligibility score from EEG signals to continuous stimuli with a linear input-output model is still a challenge. Recently, deep learning models have been widely used to classify auditory neural outcomes (Ciccarelli et al., 2019; Craik et al., 2019; Roy et al., 2019; Nogueira and Dolhopiatenko, 2020). Ciccarelli et al. (2019), showed that the non-linear model for decoding of auditory attention outperformed the linear model. As a sentence is non-linearly and hierarchically processed in the human brain along the complex auditory pathway, a non-linear model can perform better in predicting speech intelligibility. Thereby, deep learning can be successfully used in a non-linear model to investigate auditory neural processing. Deep learning requires two essential processes for better predictive performance. First, the reduction of attribute noise, which leads to a decrease in overfitting and memorization of noise data, can be achieved by neural tracking with speech features from EEG (Zhu and Wu, 2004; Altaheri et al., 2021; Cherloo et al., 2021; Zhou et al., 2021). Second, data augmentation increases the amount of data and helps to overcome the problem of limited data (Lashgari et al., 2020).

Although accurate classification is achieved through deep learning, it is essential to interpret the results for clinical use. The explainable deep learning models, the gradient-weighted class activation map (Grad-CAM), and the occlusion analysis map have been developed and applied to the classification tasks of an EEG data model (Jonas et al., 2019; Li et al., 2020; Mansour et al., 2020; Uyttenhove et al., 2020; Lombardi et al., 2021). While the Grad-CAM typically highlights the important lesion, the occlusion analysis map tracks multi-focal lesions and thus supports information with higher spatial resolution (Oh et al., 2020; Aminu et al., 2021; Govindarajan and Swaminathan, 2021). Occlusion analysis has been used to discover cortical areas related to movement tasks in EEG classification and identify important regions for image classification (Zeiler and Fergus, 2014; Ieracitano et al., 2021). In this study, we developed a deep learning model to predict speech intelligibility scores with EEG signals to continuous sentences. The typical speech features of ENV and phoneme onset impulse were used. An occlusion sensitivity map was used to select sensitive EEG channels to predict speech intelligibility scores (Esmaeilzadeh et al., 2018; Singh et al., 2020).

## Materials and methods

### Participants

Eighty-seven participants with normal hearing (44 males and 43 females) participated in this study. They were 20–33 years old (mean = 24.0 and standard deviation = 2.4). All experimental procedures and the written informed consent procedure were reviewed and approved by the Institutional Review Board of the University of Ulsan.

### Stimuli

Ten continuous sentences spoken by a male speaker were selected from the Korean standard sentence list for adults (Jang et al., 2008). The duration of each sentence was 1.8 ± 0.2 s, and the number of phonemes in each sentence was 18.6 ± 3.9. The natural (non-vocoded) and noise-vocoded sentences were used in this study. A noise vocoder was used to simulate poor sensitivity with normal-hearing listeners. The vocoder consisted of a logarithmically-spaced filter bank between 200 and 5,000 Hz. Natural sentences are then filtered through the filter bank, which is modulated with a Gaussian white noise and synthesized sequentially (Mehta and Oxenham, 2017). The channel of vocoder parameter was set to 2, 3, 4, 5, and 8 for five noise-vocoded conditions, wherein a lower number of channels generated more spectrally degraded stimuli.

### Behavioral test and electroencephalography

The Korean sentence recognition test was conducted to evaluate the behavioral speech intelligibility score in a soundproof room prior to EEG data acquisition. The test used 10 sentences selected out of 90, and the participant was asked to verbally repeat the sentence which was presented through a loudspeaker (NS-B51, YAMAHA, Hamamatsu, Japan) at a comfortable level of 60 dBA. The behavioral speech test was performed using natural and noise-vocoded sentences. The behavioral speech intelligibility score, which was calculated using the number of correctly repeated words out of 40 target words, ranged from 0 to 100, with a step of 2.5.

**FIGURE 1:**
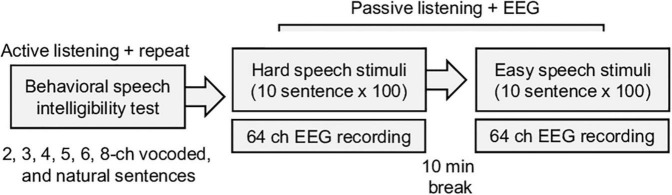
Summary of the experimental procedure for the behavior speech intelligibility test and EEG recording. During the behavioral test, vocoded noise, and natural sentence speeches are randomly played, and the participants are asked to repeat the sentences. The electroencephalogram (EEG) responses to the speech stimuli are recorded during the passive listening task.

EEG data were recorded using a 64-channel system (Biosemi Active 2, Netherlands) in a soundproof room. The natural and vocoded sentences were randomly played by a loudspeaker (NS-B51, YAMAHA, Hamamatsu, Japan) 1 m away from the participants. The inter-stimulus interval (between sentences) was set to 3 s, and each sentence was repeated 100 times. Difficult tasks had precedence over easy tasks to minimize learning throughout the tasks (Figure 1). During this passive experiment, a participant could watch a silent video with subtitles on an LCD monitor and could rest for 10 min between sessions. The raw EEG data were downsampled to 256 Hz for computational efficiency and preprocessed using the EEGLAB toolbox (Delorme and Makeig, 2004). The down-sampled EEG data were re-referenced using average referencing and band-pass (1–57 Hz) filtered by a Hamming windowed sinc FIR filter (Widmann, 2006). The typical eye-movement related artifact was rejected using the extended infomax independent component analysis and manually inspected correction. The EEG data were epoched in the intervals –0.5 to 2.5 s, relative to stimulus onset.

### Speech features: Envelope, phoneme, and envelope and phoneme

The PH onset impulse train and the ENV of the natural sentences were used as speech (stimuli) features. All PH onsets in the sentences were automatically identified (Yoon and Kang, 2013) using Praat software (University of Amsterdam, Netherlands) and manually confirmed. The number of phonemes in each sentence ranged from 17 to 22 (mean: 18.6, standard deviation: 3.9). The PH onset impulse train consisted of a sequence of unit impulses at the onset time of the phoneme. The ENVs were computed using a full-wave rectifier and a low-pass filter (30 Hz cutoff). The cutoff frequency of 30 Hz was chosen to obtain a sufficient amplitude envelope of EEG data (Souza and Rosen, 2009; Roberts et al., 2011). Using these aforementioned values, the product of the multiplication of the PH and ENV (PHENV) was calculated.

### Deep learning for speech intelligibility prediction

**FIGURE 2:**
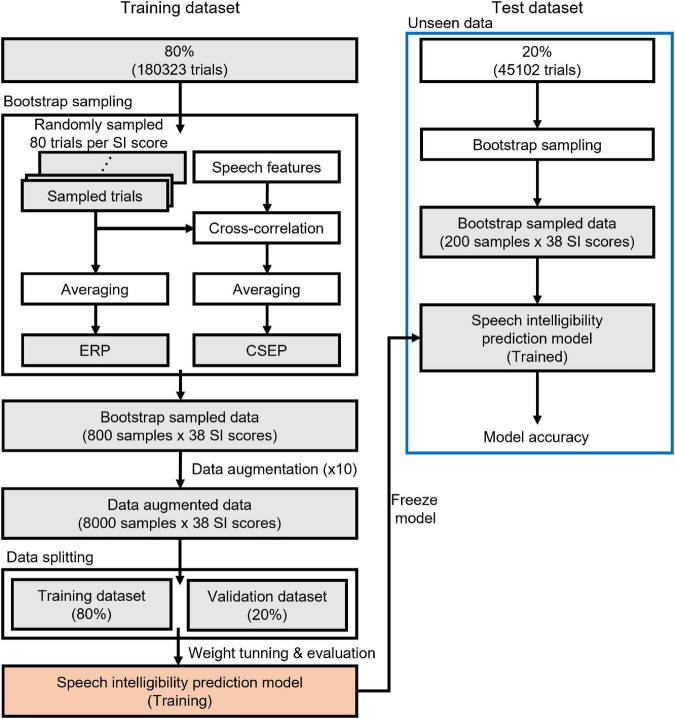
Schematic diagram of deep learning training and testing. A training dataset is used to build up a speech intelligibility prediction model and an unseen (test) dataset determines the performance of the model.

The EEG data were randomly split into a training set (80% of the original data set) and an unseen test set (20%), as depicted in Figure 2. An ERP was computed by averaging 80 EEG data epochs. A bootstrap sampling procedure was employed to generate ERPs and continuous speech-evoked potentials (CSEPs), evenly distributed across the range of speech intelligibility scores. The deep learning features of the CSEPs were computed by averaging the cross-correlation coefficients between the EEG data epochs and speech (stimuli) features. As a result, 800 ERPs and CSEPs from the training set, and 200 ERPs and CSEPs from the test set, were taken.

To enlarge the number of training datasets and guarantee that they reached 8,000, the training datasets were augmented with one of three approaches: Gaussian noise, temporal cutout, or sensor dropout. One of the approaches was randomly selected for each augmentation (Wang et al., 2018). The augmentation techniques used the best parameters obtained in a previous study (Cheng et al., 2020). Gaussian noise was added to the signal, and the ratio of noise to signal was 0.6. The temporal cutout was a random temporal window replaced with Gaussian noise, and the duration of the temporal window was 0.625 s (about 20% of the 3 s recording period). The sensor dropout was a random subset of sensors replaced with zeros, and the number of dropping sensors was 12 (about 20% of the 64 electrodes). The validation datasets (20% of the augmented training datasets) were randomly selected.

**FIGURE 3:**
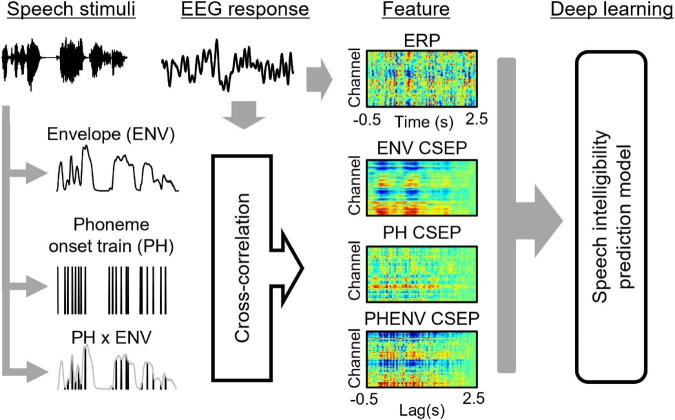
The overall scheme of speech intelligibility prediction, including speech stimuli, electroencephalogram data, feature extraction, and model prediction. The speech features of envelope (ENV), phoneme onset train (PH), and ENV ⊗ PH generate the features of ENV continuous-speech evoked potentials (CSEP), PH CSEP, and PHENV CSEP by cross-correlating with the electroencephalogram response (EEG).

Figure 3 shows the overall schematic representation of speech feature, feature extraction, and speech intelligibility classification. Four deep learning models to predict the behavioral speech intelligibility scores were trained using the ERPs, envelope-based CSEPs, phoneme-based CSEPs, and phoneme-envelope-based CSEPs. The ERP and CSEP at each channel were plotted against time after sentence onset, as seen in Figure 3. The color in each panel indicated the amplitude of the ERP and CSEP. Each panel was resized to 299 × 299 from the original size of 64 × 768 for computational efficiency, to build up the model with small kernels and numbers of layers, and then used for deep learning (Simonyan and Zisserman, 2014; Szegedy et al., 2015; He et al., 2016; Tan and Le, 2019). The deep learning architecture consisted of four convolutional layers which were fully connected. Max pooling, leakyReLU, and batch normalization layers were employed in the convolution process. See Table 1 for more details about the deep learning architecture. The Adam optimizer was used for training the deep learning models (Kingma and Ba, 2014). The initial learning rates of the optimizer, batch size, and epoch value were set to 0.001, 64, and 5, respectively. Training data were shuffled before training to avoid any bias and overfitting. Finally, four deep learning models were evaluated by computing the classification accuracy, using the unseen test set as follows:


Accuracy=(T⁢N+T⁢P)/(T⁢N+T⁢P+F⁢N+F⁢P).

**TABLE 1:** Deep learning layers and their specifications.

Deep learning layer	Filter size	Kernel dimension (H × W)	Output (H × W × D)
Input			299 × 299 × 3
Conv2D	32	16 × 16	299 × 299 × 32
LeakyReLU			299 × 299 × 32
Conv2D	8	8 × 8	299 × 299 × 8
LeakyReLU			299 × 299 × 8
Maxpooling2D		2 × 2	149 × 149 × 8
Conv2D	8	4 × 4	149 × 149 × 8
LeakyReLU			149 × 149 × 8
Maxpooling2D		2 × 2	148 × 148 × 8
Conv2D	3	3 × 3	148 × 148 × 3
LeakyReLU			148 × 148 × 3
Maxpooling2D		2 × 2	147 × 147 × 3
Batch normalization			147 × 147 × 3
Fully connected		1 × 38	1 × 1 × 38
Softmax			1 × 1 × 38
Classification			38

where *TP*, *TN*, *FP*, and *FN* denote true positive, true negative, false positive, and false negative, respectively. The occlusion sensitivity maps showed which channels contributed more to classifying the speech intelligibility score. Compromising spatial resolution and computational efficiency, the map used a 5 × 5 occluding mask and stride.

## Results

**FIGURE 4:**
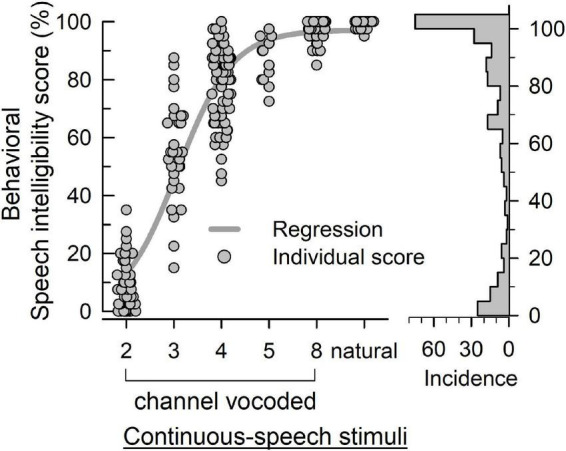
The behavioral speech intelligibility score (left panel) of individuals in response to vocoded and natural continuous-speech stimuli. The bars (right panel) indicate the incidence of each score.

Figure 4 plots the behavioral speech intelligibility scores in response to natural and noise-vocoded (2, 3, 4, 5, and 8 channel) sentences. Although the scores are not evenly distributed, it covered overall score ranges except 30.0, 37.5, and 40%. Table 2 shows the statistical summary of the behavioral scores.

**TABLE 2:** Results of behavioral speech intelligibility scores with natural and noise vocoded sentences.

Sentence type		Behavioral score (%)	
		
		Mean	*SD*
Noise vocoded	2 Channel	7.5	8.08
	3 Channel	55.2	17.03
	4 Channel	77.3	12.63
	5 Channel	86.4	8.01
	8 Channel	97.9	3.44
	Natural sentence	99.6	1.01

SD, standard deviation.

Table 3 summarizes the performance of the deep learning models. The predictive accuracies were 97.33% (ERP), 99.42% (ENV), 99.55% (PH), and 99.91% (PHENV). Compared to the probabilistic chance level of 2.63%, the four deep learning models achieved comparable performance on predicting the speech intelligibility score. The deep learning model with the feature based on the PHENV yielded the highest accuracy of 99.91%.

**TABLE 3:** Comparison of the performance of deep learning models using event-related potentials (ERP), stimuli envelopes (ENV), phonemes (PH), and phoneme-envelopes (PHENV).

	Deep learning using			
	
	ERP	ENV	PH	PHENV
Accuracy	97.33%	99.42%	99.55%	99.91%

**FIGURE 5:**
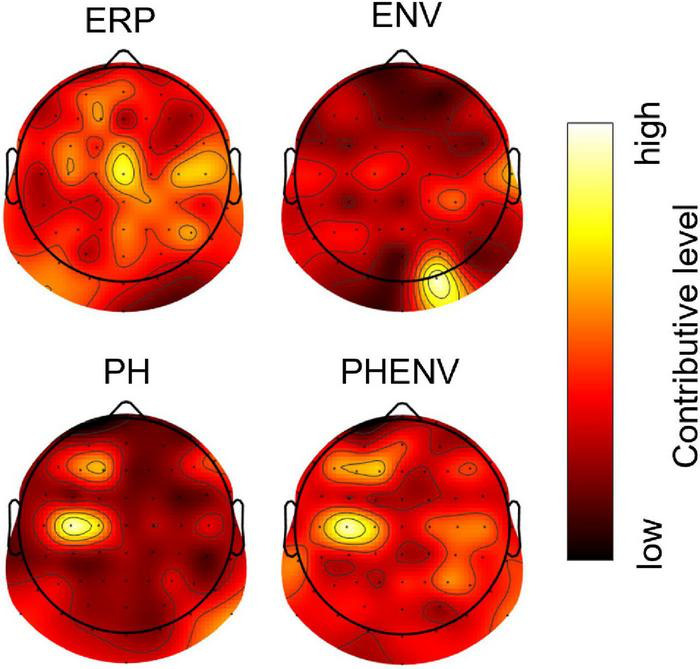
The topographic map of occlusion sensitivity visualizing the important brain regions for classification. Dark to bright red color denotes relatively low to high contributive levels for prediction.

Figure 5 shows the topographical map of occlusion sensitivity computed from the four deep learning models. The color indicates the level of contribution to a classification decision at each channel. Here, the dominant contribution was observed in the occipital region for the ENV-based model, whereas the dominancy was spread over the central, frontal, and parietal brain regions for the ERP, the PH-based model, and the PHENV-based model. Table 4 summarizes the 10 most sensitive EEG channels and the corresponding brain regions for deep learning to predict speech intelligibility scores.

**TABLE 4:** Summary of regions of significant contribution for deep learning. The ten most significant EEG channels and their regions, to predict speech intelligibility, are selected using occlusion sensitivity analysis.

Deep learning using	Regions of significant contribution for deep learning	
	
	EEG channels	Brain regions
ERP	Cz, C6, C4, FCz, P6, F1, CP2, C3, Pz, FC3	Central, frontal, parietal
ENV	O2, T8, PO4, CP4, C6, C1, T7, CP2, FT8, P5	Occipital, temporal, parietal
PH	C3, C1, F1, P10, F3, F8, P9, C5, F6, PO8	Central, frontal, parietal
PHENV	C3, C1, F1, F3, F5, P4, C5, TP7, C6, C4	Central, frontal, parietal

EEG, electroencephalography; ERP, event-related potential; ENV, stimuli envelope; PH, phoneme; PHENV, phoneme-envelope.

## Conclusion and discussion

In this study, we developed a deep learning model to predict speech intelligibility scores using continuous speech-evoked EEG signals. The cross-correlation coefficients between typical speech features (PH and ENV) and EEG responses to speech were implemented as a feature for deep learning and the model achieved the highest classification accuracy of 99.91%. The topographic map illustrating the frontal, central, and parietal regions provided important information for the classification.

Several studies have employed a linear model (i.e., TRF) to predict individual speech intelligibility from EEG responses to overlapped sentences or long story (14 min) stimuli (Lesenfants et al., 2019; Muncke et al., 2022). One issue with stimulus-driven EEG signals is the speech onset response, which is greater in magnitude than the overall neural activity. For this reason, Crosse et al. (2021) reported that the TRF model may not be a feasible approach to apply in EEG signals in response to short-duration (<5 s) stimuli (Crosse et al., 2021). It is therefore essential to consider the methodological approach to model building in response to speech and continuous stimuli. It should also be noted that the TRF model requires regularization coefficient tuning to avoid overfitting, which makes use of more computational resources and is more complex than cross-correlation. Furthermore, a deep learning model with the cross-correlation coefficient can leverage a non-linear feature to predict the non-linear property of speech intelligibility (Accou et al., 2021).

Subjects participated in the passive listening condition during the electrophysiological data collection in this study. Passive listening provides less experimental fatigue than active listening and can be performed by young children (Roy, 2018; O’Neill et al., 2019). Several studies on selective attention decoding and cortical tracking to long story stimuli have employed the active listening paradigm to keep subjects attentive (Vanthornhout et al., 2018; Lesenfants et al., 2019; Accou et al., 2021; Nogueira and Dolhopiatenko, 2022). Although these participants were asked regarding the stimuli during the experiment for active listening, it may be difficult to ensure a stable attentive level throughout the entire task. In particular, Kong et al. (2014) reported that neural responses from active and passive listeners were similar in quiet conditions, whereas the differences of cross-correlation function were observed in competing speaker conditions. Thus, attention should be considered when predicting speech intelligibility in a selective listening condition.

The occlusion sensitivity enabled the decision of deep learning interpretability (Zeiler and Fergus, 2014; Ieracitano et al., 2021). Here, occlusion sensitivity explained that neural activity from the central and left frontal region made the most important contribution to speech understanding. The topographic map of occlusion sensitivity in PH and PHENV cases showed that the language dominant region (typically F3 within the middle frontal gyrus and TP7 within the middle temporal gyrus) was highly involved in speech intelligibility processes (Scrivener and Reader, 2022). The results are comparable with the findings of neuroimaging studies, specifically that of the sentence-processing network, including the middle frontal and middle temporal gyri (Peelle et al., 2004, 2010; Fiebach et al., 2005; Smirnov et al., 2014). Also, it supports the middle temporal gyrus and the supramarginal gyrus involvement in syntactic and phonological processing (Friederici, 2011). Deep learning with PH and PHENV could be reasonably explainable and interpretable by occlusion sensitivity.

This study has several limitations for clinical implementation. The deep learning model was developed using data from a limited group. The noise-vocoder was used to simulate hearing impairment with normal hearing listeners. Since no data from cochlear implant and hearing aid users were accessed, the model should be sufficiently validated with data of hearing-impaired individuals. In addition, although the group-level deep learning model was developed and tested in this study, it was still challenging to optimize the model with individual-level features due to inter-subject variability (Cheng et al., 2020; Accou et al., 2021). Further investigation of subject-specific models is necessary for the clinical prediction of speech intelligibility. These are the key issues for future studies. We also plan to improve the model by incorporating source EEG data rather than 64-channel EEG data and optimizing the channels based on the occlusion sensitivity map.

## Data availability statement

The data used to support the findings of this study are available from the corresponding author upon request.

## Ethics statement

The studies involving human participants were reviewed and approved by the Institutional Review Board of the University of Ulsan. The patients/participants provided their written informed consent to participate in this study.

## Author contributions

YN and JW designed the experiment, developed the model, and examined the results. YN, LT, HJ, and LQ collected data and performed data preprocessing. All authors were involved in preparing the manuscript.
